# Versatile Types of Polysaccharide-Based Drug Delivery Systems: From Strategic Design to Cancer Therapy

**DOI:** 10.3390/ijms21239159

**Published:** 2020-12-01

**Authors:** Yanzhen Sun, Xiaodong Jing, Xiaoli Ma, Yinglong Feng, Hao Hu

**Affiliations:** 1 Institute of Biomedical Materials and Engineering, College of Materials Science and Engineering, Qingdao University, Qingdao 266071, China; sunyanzhen1920@163.com (Y.S.); Jingxiaodong5230@163.com (X.J.); fengyinglong1215@163.com (Y.F.); 2 Qingdao Institute of Measurement Technology, Qingdao 266000, China; maxiaoli1989@yeah.net

**Keywords:** polysaccharide, drug delivery system (DDS), chemotherapy, cancer therapy

## Abstract

Chemotherapy is still the most direct and effective means of cancer therapy nowadays. The proposal of drug delivery systems (DDSs) has effectively improved many shortcomings of traditional chemotherapy drugs. The technical support of DDSs lies in their excellent material properties. Polysaccharides include a series of natural polymers, such as chitosan, hyaluronic acid, and alginic acid. These polysaccharides have good biocompatibility and degradability, and they are easily chemical modified. Therefore, polysaccharides are ideal candidate materials to construct DDSs, and their clinical application prospects have been favored by researchers. On the basis of versatile types of polysaccharides, this review elaborates their applications from strategic design to cancer therapy. The construction and modification methods of polysaccharide-based DDSs are specifically explained, and the latest research progress of polysaccharide-based DDSs in cancer therapy are also summarized. The purpose of this review is to provide a reference for the design and preparation of polysaccharide-based DDSs with excellent performance.

## 1. Introduction

As a systemic treatment method, chemotherapy is still the most commonly used method for cancer therapy. Chemical drugs can be used in chemotherapy to block the proliferation, infiltration, and metastasis of tumor cells, thereby killing cancer cells [1]. However, there are still several challenges to make chemotherapy drugs work smoothly in tumor tissues and cells: (i) short blood circulation time and poor water solubility make the bioavailability of small molecule drugs low; (ii) long-term medication makes tumor cells resistant to chemotherapy drugs; (iii) chemotherapy drug molecules cannot specifically reach the tumor site and penetrate normal tissues indistinguishably, leading to nausea, vomiting, diarrhea, constipation, alopecia, and decreased liver function, which aggravates the suffering of patients. Fortunately, with the rapid development of nanotechnology, new nanomedicine technologies that are applied to the field of cancer treatment have gradually emerged. By embedding, adsorbing, or covalently coupling traditional chemotherapeutic drug molecules onto/into a nano-sized carrier, researchers have proposed drug delivery systems (DDSs) [2]. Through various functional modifications to the carrier, this system has the advantages that traditional drug molecules cannot match. Compared with traditional drug molecules, DDSs possess better water solubility and stability, higher specific delivery in vivo, lower toxic side effects, and higher anti-tumor effect [3]. At present, a wide variety of DDSs have been developed and applied, such as proteins, polysaccharides, synthetic polymers, and organo–inorganic hybrids [4,5,6,7]. Among them, the polysaccharide is a natural polymer formed by dehydrative condensation of multiple monosaccharide molecules [8]. The polysaccharides commonly used in the biomedical materials field include hyaluronic acid [9], chitosan [10], dextran [11], alginate [12], and chondroitin sulfate [13]. These polysaccharides are safe and non-toxic, easily biodegradable and chemically modified, and have good water solubility and biological activity (such as anti-tumor, anti-viral, anti-oxidation, and anti-inflammatory effects, as well as prevention of various cardiovascular diseases). Moreover, large reserves and low production costs make polysaccharide products easy to produce on a large scale.

Polysaccharides are rich in sources and can be obtained from various natural renewable resources (ocean and plant). A lot of functional groups such as hydroxyl (-OH), amino (-NH_2_), or carboxyl (-COOH) hang on the molecular chain of polysaccharide. The presence of these groups makes it easy for polysaccharides to be chemically modified [14]. By changing the physical and chemical properties of polysaccharides, structures such as vesicles, micelles, and hydrogels are formed. In recent years, a series of controllable polymer-grafted polysaccharides are made by living radical polymerization (LRP) and ring-opening polymerization (ROP) [15,16]. These modified polysaccharides have a great application prospect in drug delivery, gene delivery, and wound dressing [17,18,19,20]. Liposomes, micelles, and hydrogels modified by polysaccharides have been widely used as excellent anti-tumor DDSs [21,22,23]. While maintaining the advantages of polysaccharide materials, such DDSs also possess various advantages of nanoscale systems. Polysaccharides are one of the candidates for constructing hydrogels and have been extensively studied. Polysaccharide-based hydrogels loaded with drugs or bioactive molecules are widely used as wound dressings, and many products have been commercialized [24]. In situ injectable polysaccharide-based hydrogel is a good local sustained-release carrier and may be able to isolate tumors from normal tissues [25,26].

In view of the excellent performance of polysaccharide-based DDSs, this article presents the overall situation of versatile types of polysaccharide-based DDSs for cancer therapy. The design strategies and considerations about polysaccharide-based DDSs are discussed, and the advanced research progress in the field of tumor therapy are summarized. Finally, we outline the research prospects and application value of polysaccharide-based DDSs.

## 2. Design Strategies

**Figure 1:**
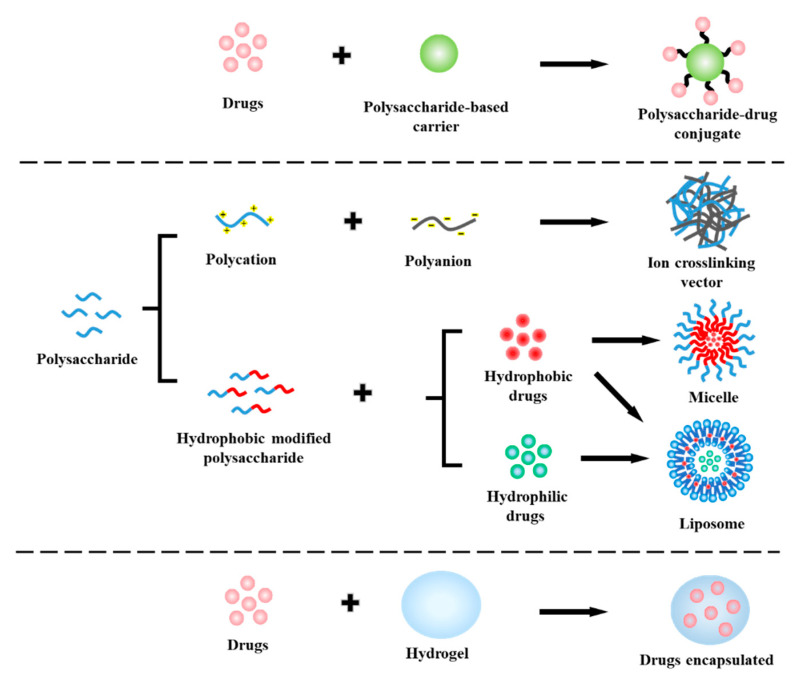
The design strategies of polysaccharide-based drug delivery systems (DDSs).

Polysaccharides are composed of monosaccharides, including *D*-glucose, *D*-galactose, *D*-fructose, *D*-mannose, *D*-xylose, *L*-galactose, and *L*-arabinose, which are combined by glycosidic bonds [27]. Polysaccharides are mainly derived from various natural products, such as alginic acid from algae, pectin and guar gum from plants, dextran and xanthan gum from microorganisms, and chitosan and chondroitin from animals [28]. Polysaccharide materials derived from nature not only have inherent environmental friendliness, but also adapt well to cell physiology. Drug carriers have been widely studied as auxiliary materials for the delivery of chemotherapeutics. Inorganic nanoparticles, liposomes, micelles, and hydrogels are currently the most commonly used drug systems [29]. By modifying polysaccharide molecules, derivatives with different functional groups and conjugates can be obtained, which is conducive to further synthesis of drug carriers. In order to reduce the side effects during chemotherapy and prevent the excessive use of drugs, it is of great significance to study how to combine polysaccharides with drugs to construct DDS. For polysaccharide-based drug delivery systems, the design strategies can be broadly divided into three types: (i) coupling polysaccharides and drugs through cleavable chemical bonds to construct polysaccharide–drug conjugate carriers [30]; (ii) self-assembling to form polysaccharide-based drug-loaded particles [31]; (iii) encapsulating drug molecules in polysaccharide-based hydrogels [32]. The common design strategies of polysaccharide-based DDS are shown in Figure 1.

### 2.1. Constructing Polysaccharide–Drug Conjugates

Natural or synthetic water-soluble polymers are usually applied to drug coupling to develop various prodrugs [33]. The composition of monosaccharides is diversified, with neutral hydroxyl groups, negatively charged carboxylic acid/sulfate groups, or positively charged amine groups [34]. Connections between monosaccharide units are variable (such as linear chain structure and branched chain structure), and there are also great changes in molecular weight. These complex chemical connections provide the basis for the huge chemical diversity of polysaccharides. Hydroxyl (-OH), carboxyl (-COOH), and amino (-NH_2_) on the polysaccharide molecules are the main active sites used to construct polysaccharide-based DDS. Polysaccharides can form polysaccharide–drug carriers through non-covalent interactions or covalent linkage reactions with drug molecules. Common construction methods include (i) esterification of hydroxyl groups with acylating agents, etherification of hydroxyl groups with alkylation agents, oxidation of primary alcohols to carboxyl groups, and oxidation of vicinal secondary hydroxyl groups to aldehydes; (ii) ester bonds consisting of hydroxyl groups linked to carboxyl groups, amide bonds consisting of carboxyl groups linked to amino groups, and hydrazone bond formed by reaction of -COOH and -NHNH_2_; (iii) interaction between amino groups and hydroxyl or carboxyl groups [35].

### 2.2. Self-Assembling Polysaccharide-Based DDS

#### 2.2.1. Cross-Linking between Polymeric Electrolyte and Ion

Charged polysaccharides can be cross-linked through interaction with oppositely charged polymers or simple ions. The polymeric electrolyte self-assembly system of polysaccharides is mainly based on the mutual attraction of positively charged chitosan (or chitosan oligomers) and negatively charged polyanionic polysaccharides (including carboxymethyl cellulose [36], carboxymethyl glucomannan [37], carboxymethyl chitosan (CMC), dextran sulfate [38], alginate [39], heparin [40], octenyl succinic anhydride starch (OSA starch) [41], carboxymethyl starch, and hyaluronic acid [42]). The hydrogel constructed by ionic crosslinking can be adapted to pH-controlled drug release by adjusting swelling and dissolution [43]. For example, alginate can chelate polyvalent metal ions (such as calcium ions) to form nano-scale gels. These gels have been widely used as sustained DDS due to their excellent biocompatibility and non-immunogenicity [44]. Carboxymethylated chitosan can be transformed into nanoparticles by interacting with calcium ions. The particle size distribution can be controlled by adjusting the addition parameters of calcium ions [45].

#### 2.2.2. Self-Assembly of Hydrophobic Polysaccharides

Micelle is a kind of widely used nanocarrier. The polymers forming micelles have hydrophilic and hydrophobic moieties, which can self-assemble into micelles in aqueous solution to achieve solubilization and encapsulation of drugs. The hydrophobic core facilitates the micelles to carry drugs with different properties; the hydrophilic shell helps drug-loaded micelles to escape the phagocytosis of mononuclear phagocytes. Polysaccharide molecules themselves are not amphiphilic and cannot form polymer micelles through self-assembly. By introducing hydrophobic molecules into the hydrophilic polysaccharide backbones, the resulting amphiphilic polysaccharide molecules have the ability to self-assemble into nanoscale micelles in aqueous solution [46]. Polymer liposome is a kind of spherical molecular aggregate formed by self-assembly of amphiphilic polymers. It has a bilayer membrane structure similar to human cell membranes. The preparation of polysaccharide-composed liposomes is similar to that of polysaccharide-based micelles, and the hydrophobic modification of polysaccharide molecules is also required [47]. Hydrophobic molecules such as cholesterol, steroid acid, deoxycholic acid, and hydrophobic polymers are commonly used to modify polysaccharide molecules. They allow polysaccharide-based amphiphilic polymers to self-assemble into micelles or liposomes by minimizing interface free energy [48]. Hydroxyl, amino, and carboxyl groups on the hydrophilic polysaccharide skeleton are commonly used sites for hydrophobic grafting. Liposomes have the ability to simultaneously carry hydrophilic drugs and hydrophobic drugs. The unique bilayer structure of liposomes allows both hydrophobic and hydrophilic microdomains to exist. The water-soluble drugs are encapsulated in the hydrophilic cavity of the liposome, while the hydrophobic drugs are located in the hydrophobic microphase between the two phospholipid bilayers of the liposome. The controlled release of drugs can be realized according to the change of microenvironment (temperature, pH, etc.) [49].

### 2.3. Preparation of Drug-Loaded Polysaccharide-Based Hydrogels

Hydrogel is a kind of polymer with three-dimensional network structure [50]. With high water content and hydrophilicity [51], the hydrogel can quickly inhale and store water far exceeding its own volume [52]. Polysaccharides are good candidates for the preparation of drug-loaded polysaccharide-based hydrogels. Nanoscale gel particles also have great advantages in the controlled release of anti-tumor drugs [53]. Drug molecules can be encapsulated in the matrix of the gel through non-covalent interactions (such as salt bond, hydrogen bonds, and hydrophobic interaction), and the drug can be released from the gel through the expansion of the grid [54]. Some prodrug molecules can also self-assemble into hydrogels to realize self-delivery and sustained release [55]. For instance, after encapsulating the chemotherapy drug doxorubicin (DOX), the hydrogel was injected into tumor tissues, which could isolate tumor cells and achieve long-term drug release in local tumor [56].

## 3. Functionalization of Polysaccharide Molecules

In order for researchers to achieve targeted drug delivery and precise control of drug release behavior in tumor lesion tissues, it is of great significance to construct functional DDS that respond to the tumor microenvironment in order to achieve active targeting or self-degradation functions. Considering that polysaccharide-based carriers can be non-specifically recognized and adsorbed by plasma proteins, allowing selective uptake of the carrier by the reticuloendothelial system results in a decrease in the bioavailability of the carrier and limiting the therapeutic effect of the drug. Therefore, it is necessary to modify the structure of the polysaccharide molecule, constructing the carrier so that it achieves specific recognition and adhesion to tumor tissue. The introduction of different functional groups to modify polysaccharides (such as in situ disulfide bond modification) during the construction of polysaccharide-based carriers can make these carriers environmentally responsive. By responding to the stimulation of the tumor microenvironment (pH, glutathione (GSH), etc.), the biodegradation rate of the carrier is changed, and then the drug release rate is adjusted. The commonly used functionalized types of polysaccharide molecules are summarized in Table 1.

### 3.1. Modification of Functional Molecules on Polysaccharide Molecules

**Figure 2:**
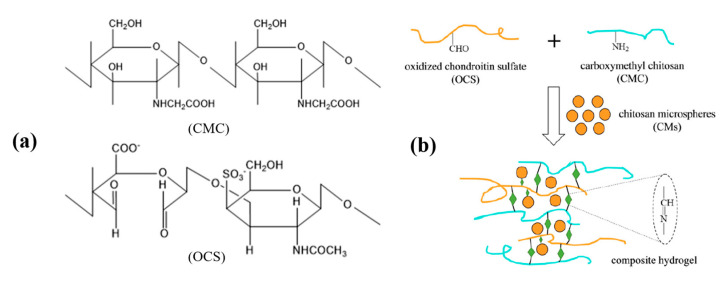
(**a**) Chemical structures of carboxymethyl chitosan (CMC) and oxidized chondroitin sulfate (OCS). (**b**) Reaction scheme to show a preparation of chitosan-based microspheres (CMs) embedded in CMC–OCS composite gel scaffold (CMs/gel). Modified from [21].

By modifying tumor cell-specific recognition ligands (such as antibodies, polypeptides, small molecules, and aptamers) on polysaccharide molecules, researchers endow polysaccharide-based DDSs with active tumor targeting, thereby improving the choice of anti-tumor drugs in vivo and enhancing the anti-tumor effect. For example, after being modified with folic acid molecules (FA), the targeting ability of chitosan-composed micelle was improved [57]. When chitosan is attached with DOX, an amphiphilic polymer is formed, which can form micelles in solution. Then, the constructed DDS has nuclear targeting function after being modified with FA. In vivo experiments proved that the chitosan-composed micelle could be an ideal DDS for tumor therapy [58]. If the polysaccharide molecular structure contains a large number of ortho hydroxyl groups, periodate can be used to oxidize the ortho hydroxyl groups to aldehyde groups. Aldehyde groups can be further derivatized and convert into reversible covalent bonds such as imines, oximes, acetals, and hydrazones [65]. Aldehydes and ketone groups can undergo condensation reactions with amine compounds to form Schiff base structures, which can be degraded rapidly in an acid environment. Therefore, aldehyde modification is a common method to prepare pH-responsive polysaccharide-based DDS [66]. Fan et al. prepared a covalent and injectable chitosan/chondroitin sulfate hydrogel embedded with chitosan microspheres for drug delivery and tissue engineering. The chondroitin sulfate was oxidized to oxidized chondroitin sulfate (OCS) first and then reacted with CMC via Schiff base cross-linking reaction to form hydrogels. Bovine serum albumin (BSA)-loaded chitosan-based microspheres (CMs) were encapsulated in the hydrogel as an active component (Figure 2) [21].

### 3.2. Molecular Chain Grafting

**Figure 3:**
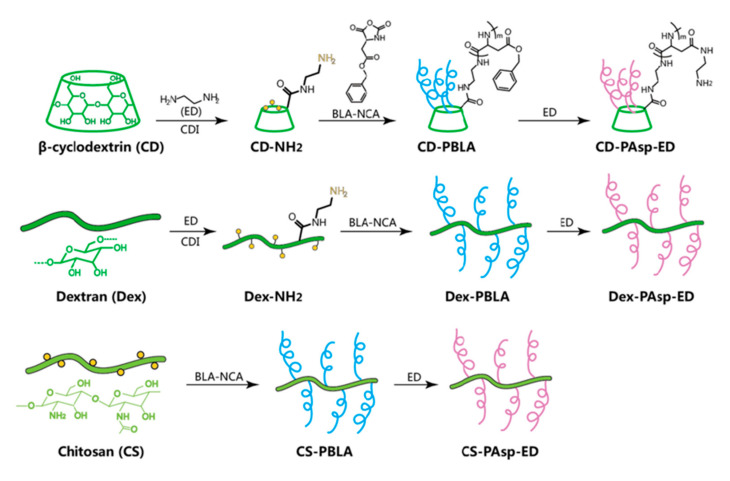
Illustration of the preparation processes of several graftable polysaccharides. Modified from [70].

The chemical properties of a polymer can be controlled by grafting side chains. The grafted chains can be controlled precisely by using the free radical polymerization method. Meanwhile, the branched chains introduced by covalent bonds have long-term chemical stability [67]. The amphiphilic graft polymer with high graft density and narrow molecular weight distribution can be obtained by modifying the hydrophobic polymer chain segment on the polysaccharide molecule. This method usually requires the use of active groups on the polysaccharide molecular chain to initiate polymerization of polymer monomers. Commonly used polymerization reactions include ROP and controlled living polymerization (CRP) [68]. For example, hydrophobic polymers are grafted onto polysaccharides by initiating the polymerization of cyclic monomers using hydroxyl groups in the side chains of polysaccharides. The resulting amphiphilic polysaccharides can self-assemble to form micelles or liposomes. Currently, commonly used graft polymers include poly(lactic acid) (PLA), polycaprolactone (PCL), and polyglycolide acid (PGA) [69]. Hu et al. explored different strategies to synthesize and design controllable polymer-grafted polysaccharides. Through the utilization of LRP, the functionalization of polysaccharides was realized flexibly and effectively. The grafted substances introduced include cationic components for nucleic acid delivery, polyethylene glycol and amphiphilic ions for shielding effects, and polymers for bioimaging and bioactive drug release. Biodegradable polymer-grafted polysaccharides were prepared by ROP. A series of poly(amino acid)-grafted polysaccharides (linear, star-shaped, and comb-shaped copolymers) with advanced structures were proposed [59]. Several polymer-grafted polysaccharides are shown in Figure 3 [70]. However, direct modification methods are not easy to control the structure and relative molecular mass of polymer segments. Therefore, it has also been reported that polymeric hydrophobic segments were first synthesized and then grafted onto polysaccharide molecules after modification by active groups [60]. Compared with polysaccharides modified by polymeric hydrophobic chain, the polysaccharide molecules modified with the hydrophobic chain of small molecule possess more uniform chain length and can more accurately determine the molecular weight of the grafted polymer. Currently, small molecules commonly used for grafting polysaccharides are lauric acid [71], palmitic acid [72], and stearic acid [73]. Taking polysaccharide molecule as the skeleton, the reaction between the activated small molecule chain and the group on the polysaccharide molecule chain can obtain an amphiphilic polysaccharide with a comb-like structure [61].

### 3.3. Introduction of Cleavable Bonds

**Figure 4:**
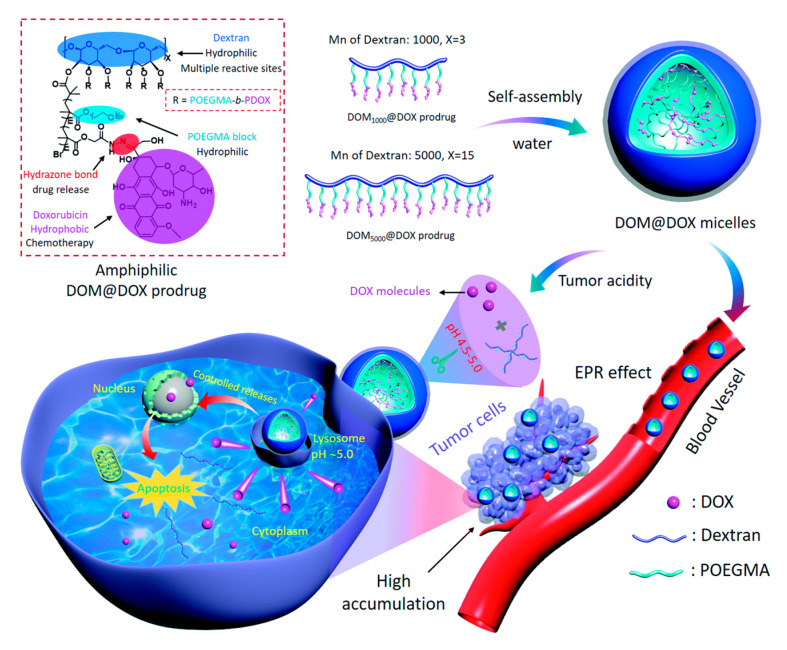
Schematic illustration of the synthetic route of acid-activated supramolecular nanoprodrug (DOM@DOX) micelles, drug accumulation via the enhanced permeability and retention (EPR) effect, cell internalization process, and pH-responsive drug release mechanism. Taken from [64].

Stimuli-responsive DDSs can respond to special microenvironmental states of tumors (weak acidity, high reductivity, etc.) with changes in structure, size, or shape to overcome physiological barriers during drug delivery [74]. These changes are usually caused by changes in the structure of the polymer. By introducing the stimuli-responsive cleavable bonds into the polysaccharide molecules, the polysaccharide-based DDS can be endowed with the ability to change its structure in response to a special environment [75]. Disulfide bonds, which can be rapidly cleaved by GSH, are commonly used to construct reduction-responsive DDSs. Polysaccharide itself does not contain disulfide bond functional groups, and thus the key to introducing disulfide bond functional groups into polysaccharide-based DDSs is to make them exist in the crosslinking agents. Generally, substances with disulfide bonds (e.g., cysteamine dihydrochloride (CYS)) are selected as cross-linking agents to enable DDS to have redox response ability. Liu et al. prepared a cellulose hydrogel by mixing cellulose acetoacetate and CYS in an aqueous solution. As a cross-linking agent, CYS introduced disulfide bond functional groups into the DDS, enabling the DDS to have redox response ability [62]. However, the types of such crosslinking agents are limited. Therefore, it is meaningful to prepare crosslinking agents containing disulfide bonds to introduce the disulfide bonds into the DDS. Yang et al. first prepared the hydrophilic skeleton by modifying dextran with succinic acid. *L*-phenylalanine ethyl ester hydrochloride (*L*-Phe) and *L*-cysteine ethyl ester hydrochloride (*L*-Cys) were used as hydrophobic groups and crosslinking points. The prepared polymer could self-assemble into micelles and load DOX hydrochloride. At the same time, the hydrosulphonyl could be oxidized into disulfide bond. Therefore, this nanoparticle had dual pH/redox response capabilities [63]. The introduction of acid-sensitive cleavable bonds can make polysaccharide DDSs have a pH-responsive ability. Zhang et al. designed an acid-activated supramolecular nanoprodrug (DOM@DOX) on the basis of dextran. As shown in Figure 4, the prodrug molecule was connected to hydrophilic polyethylene glycol through atom transfer radical polymerization (ATRP), and DOX was bound to the main chain of the copolymer using hydrazone bonds. Through the self-assembly strategy, the synthesized carrier had the ability to maintain a stable micellar structure in aqueous solution. DOX was released from micelles by breaking the hydrazone bonds in a slightly acidic environment [64].

## 4. Advances of Polysaccharide-Based DDSs

**Figure 5:**
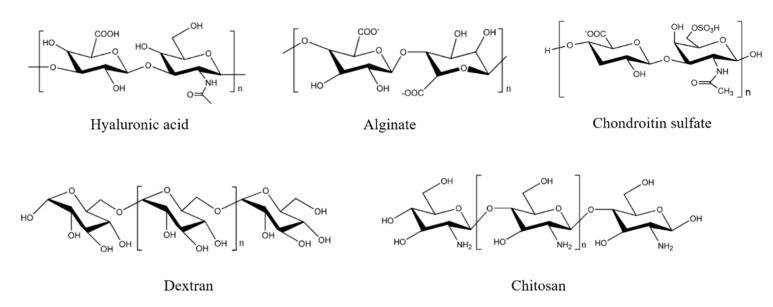
Structures of representative polysaccharides.

Many polysaccharides and their derivatives have been explored and applied in the research of anti-tumor drug delivery carriers. Hyaluronic acid, chitosan, dextran, alginate, and chondroitin sulfate (Figure 5) are commonly used materials for constructing delivery systems. These polysaccharides not only have the excellent properties, but also give the carrier special functions during the carrier construction process, such as tumor target of hyaluronic acid and chondroitin sulfate, and bioadhesion of chitosan and alginate [76]. The following section focuses on the advances of polysaccharide-based DDS.

### 4.1. Hyaluronic Acid

**Figure 6:**
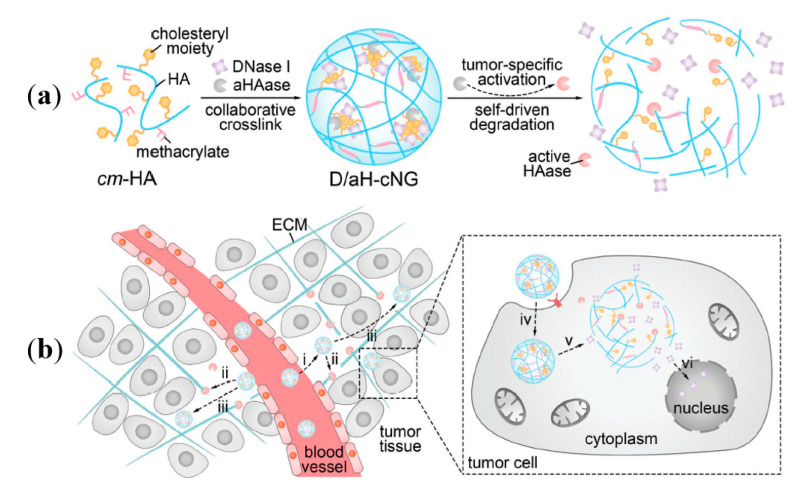
(**a**) Schematic of self-assembly and tumor-specific self-degradation of the collaboratively crosslinked crosslinked nanogels (cNG). (**b**) Schematic of enhanced protein delivery by the cNG for cancer therapy. Modified from [84].

Hyaluronic acid (HA) is a non-sulfated glycosaminoglycan that mainly exists in vivo as sodium hyaluronate, which is formed alternately by *D*-glucuronic acid and *N*-acetyl-D-glucosamine [77]. HA is an important component of the extracellular matrix and is related to the movement and proliferation of cells [78]. In the field of tumor drug delivery, HA derived from the organism itself has low immunogenicity. More importantly, it can bind to the upregulated cluster of differentiation 44 (CD44) receptor in cancer cells and mediate the biological behavior of cells [79]. In a low-pH environment (such as lysosomes), HA could be degraded [80]. The degradation products can bind to macrophages or dendritic cell receptors, thereby activating the body’s innate immunity [81]. HA could be easily chemically modified by esterification reaction and peptide-coupling reaction, and has been used for targeted drug delivery of paclitaxel drugs (e.g., paclitaxel (PTX) and DOX) [82,83]. A recently reported tumor-specific self-degradable nanogel based on HA could be used for efficient delivery of intravenously administered protein drugs (Figure 6). Using a hyaluronic acid derivative named synthetic cholesteryl-6-aminohexylcarbamate methacrylated hyaluronic acid (cm-HA) as the matrix, researchers assembled collaboratively crosslinked nanogels (cNG) by using the hydrophobic interaction between cholesterol groups for physical crosslinking and radical polymerization of methacrylate groups for chemical crosslinking. In the weak acid environment of the tumor site, acid-activatable HAase (aHAase) in cNG was partially released, which degraded HA in the extracellular matrix and promoted the diffusion of the carrier in the matrix. After endocytosis, aHAase was fully activated by acid lysosomes, the cNG was degraded by activated aHAase, and then the protein drugs contained in cNG were released and exerted anti-tumor effects [84]. Li et al. prepared HA-based nanoparticles (HA-NPs) via ion pairing between positively charged DOX and negatively charged HA. Subsequently, HA-NPs were encapsulated in liposome carriers to obtain DOX-loaded HA-based liposomes (HA-LPs) with sustained release effects. The presence of HA made the carrier become tumor-targeting, significantly increased the blood circulation time of DOX, decreased the accumulation of DOX in normal tissues, and reduced the toxicity of DOX to vital organs of the body [85].

### 4.2. Chitosan

**Figure 7:**
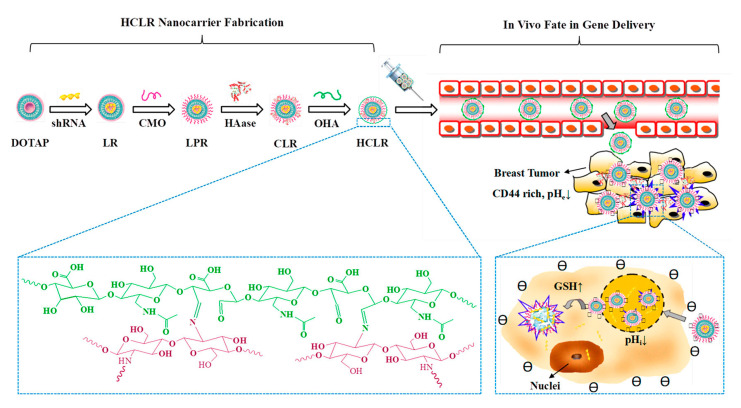
Schematic of HCLR nanocarrier fabrication and the in vivo fate in breast tumor targeting gene delivery. Taken from [92].

Chitosan (CS) is an important derivative of the natural polysaccharide chitin [86]. It is a natural cationic polysaccharide containing amino, which possess good biocompatibility. The presence of amino groups makes chitosan easy to be chemically modified. CS-based DDSs have low immunogenicity and are endowed with the ability to adhere to the negatively charged cell membrane, which is conducive to cell endocytosis [87]. Although CS has many advantages in drug delivery, its water solubility is insufficient. Therefore, it is often modified by hydrophilic groups (such as carboxymethyl and quaternary ammonium salt groups). CS can be converted to CMC by carboxymethylation under the condition of monochloroacetic acid. Compared with CS, the water solubility, biocompatibility, antibacterial activity, and biodegradability of CMC are improved. CMC has great development potential in the field of drug delivery [88]. Chi et al. prepared a series of norcantharidin (NCTD)-conjugated CMC conjugates (CNC) by using CMC as the skeleton [89]. CNC was found to have a long blood circulation time in vivo, have low distribution in the kidney and heart, and could effectively inhibit the proliferation and migration of cancer cells. Wang et al. constructed a new type of DDS by loading galactosylated chitosan (GC) onto the graphene oxide (GO). The loading capacity of DOX of this DDS was up to 1.08 mg/mg (drug per polymer). The existence of GC enabled the carrier to remain stable under physiological conditions while achieving drug release in a low pH environment [90]. CS-based DDSs can also be optimized in combination with physical methods. For example, by inserting the layered rectorite (REC) into the amphiphilic CMC chains, the prepared nanocarrier has a compact structure. The introduction of the REC layer enables the carrier to effectively capture DOX. This chitosan-based carrier can not only effectively increase the loading capacity of DOX, but also exhibit pH sensitivity to prevent the burst release of the payloads [91]. The presence of CS can promote the release of genes into the nucleus for enhancing the transfection efficiency of genes. Chen et al. deposited the modified redox-sensitive amphiphilic CS and HA layer-by-layer on liposomes to obtain a new type of continuous stimuli-responsive CS-encapsulated nanodrug delivery system (-HA/HAase/CS/liposome/shRNA (HCLR)). HCLR had the ability to stably exist in the blood circulation and to target and identify tumor sites, and was able to achieve the delivery of the silence the inhibitor of apoptosis (IAP) inhibitor survivin-shRNA gene to the nucleus (Figure 7) [92].

### 4.3. Dextran

**Figure 8:**
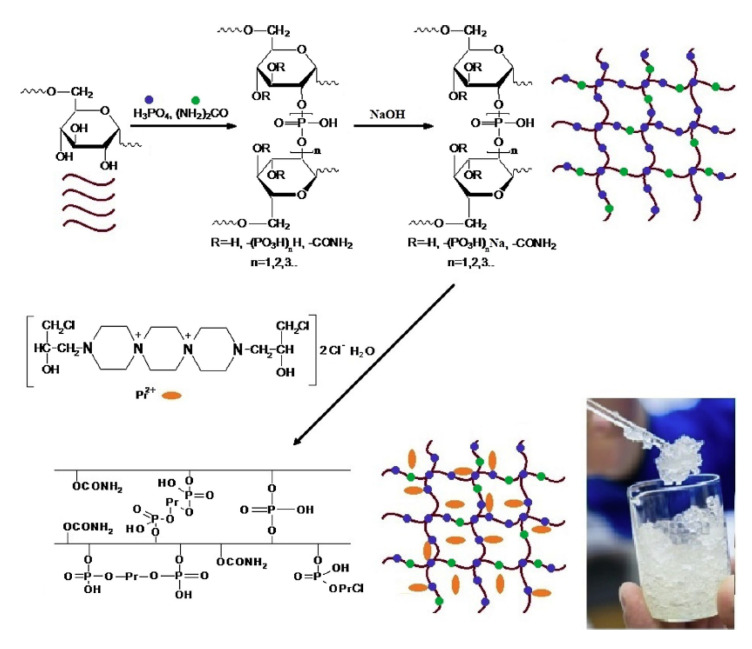
Schematic illustration of the synthesis of dextran phosphate (DP)–prospidine (Pr) hydrogels. Taken from [101].

Dextran, also known as glucan, is a water-soluble polysaccharide compound similar to starch. It is widely used in the adjuvant treatment of diseases such as hemorrhagic shock and thrombus. Dextran has colloidal properties, good hydrophilicity and water solubility, and is inert in organisms. The molecular chain of dextran is rich in hydroxyl groups, which is convenient for chemical modification. These properties provide favorable conditions for the construction of dextran-based DDSs. Dextran has been used to construct carriers of hydrogels [93], micelles [94], and core–shell structures [95] for in vivo delivery of siRNA and protein drugs. Such carriers can achieve controlled release of drugs and avoid the inactivation of drugs in the body due to the action of body fluids and enzymes [96]. The core–shell-structured carrier prepared by poly(*DL*-lactide-*co*-glycolide) (PLGA)-grafted modified dextran showed a fast drug release rate in the presence of dextranase [97]. Li et al. used ovalbumin to simulate antigens and grafted modified ovalbumin onto cationic dextran through disulfide bonds. The prepared dextran-based nanogels can stably exist in the extracellular matrix. When the concentration of GSH increased, the grafted ovalbumin was released in response. This study verified the possibility of dextran nanogels being transported in target cells as antigenic drug carriers [98]. Recently, a dextran-based conductive hydrogel was reported as a drug delivery system. The dextran was modified by electroactive aniline trimer so that the hydrogel possessed stable rheological property and a controllable swelling ratio. Therefore, the release of drugs can also be precisely controlled by electrical stimulation [99]. Wu et al. designed a carboxybetaine-modified dextran-polycaprolactone (CB-Dex-PCL) micelle for biocompatible drug carriers. The results showed that the prepared drug carrier has good protein antifouling function and could effectively prolong the circulation time of drugs in the blood [100]. Dextran phosphate (DP) has high adsorption capacity and could react with biologically active molecules. Solomevich et al. successfully prepared a pH-sensitive and biodegradable DP hydrogel. After encapsulating prospidine (Pr), it could be used in controllable DDSs (Figure 8) [101].

### 4.4. Alginate

**Figure 9:**
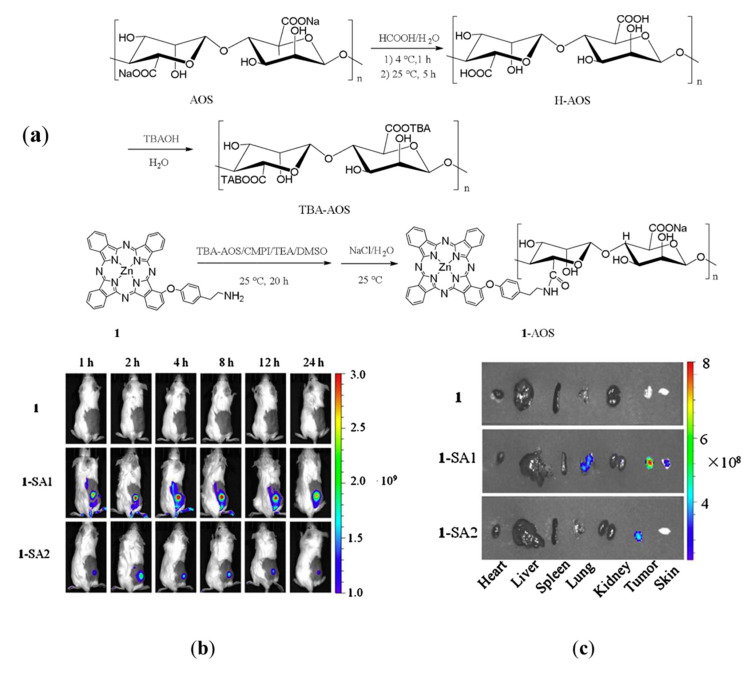
(**a**) Schematic diagram of the design and synthesis of a novel conjugation vehicle of a photosensitizer based on sodium alginate (SA). (**b**) In vivo fluorescence images of tumor-bearing KM mice after intravenous injection of SA-based carriers with different molecular weights. The red area represents tumor sites. (**c**) Ex vivo fluorescence images of organs and tumor at 24 h after injection of SA-based carriers with different molecular weights (1-SA1 is low-molecular-weight SA-based carriers). Modified from [106].

Alginic acid is mainly derived from the cell walls of brown algae [102]. The unique egg box structure and chemical properties make it easy to form gels in aqueous solution. Alginate can form hydrogel through intermolecular and intramolecular crosslinking with divalent metal ions (Ca^2+^, Cu^2+^, Zn^2+^, Pb^2+^, etc.) in aqueous solution [103]. The negatively charged alginate can also combine with cationic drug molecules through electrostatic interaction. Alginate nanoparticles treated with hydrophobic molecules such as poorly hydrophobic CS can form hydrophobic nanocarriers for drug delivery in tumor tissues while maintaining biocompatibility and low cytotoxicity [104]. For example, alginate-based amphiphilic core–shell nanoparticles can be used for the targeted delivery of DOX [105]. Tang et al. prepared a phthalocyanine (the second-generation photosensitizer (Pcs)) coupling carrier by using low molecular weight sodium alginate (SA). SA, as an anionic polymer, could bind to the highly expressed receptors (SR-A) on tumor-associated macrophages, allowing the Pcs to target tumors. Meanwhile, SA could significantly promote the dissolution of hydrophobic Pcs in aqueous solution and does not require any surfactants, which greatly improves the treatment efficiency of photodynamic therapy (Figure 9) [106].

### 4.5. Chondroitin Sulfate

**Figure 10:**
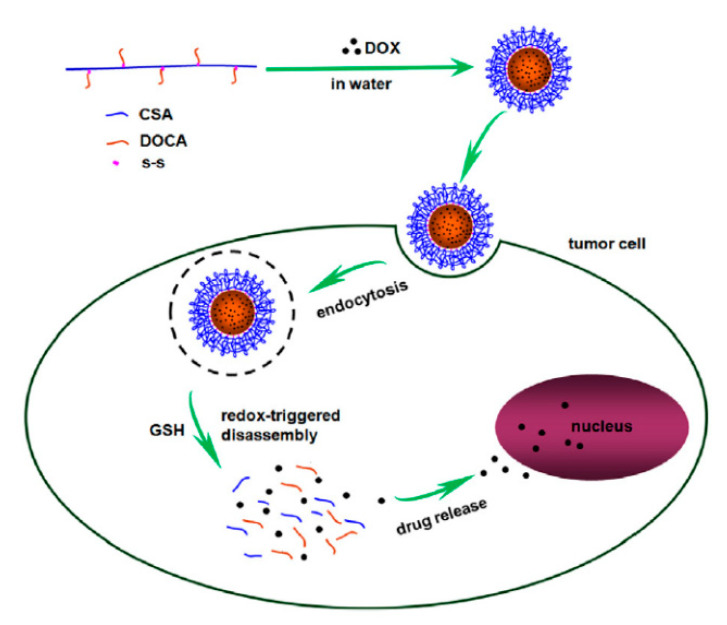
Illustration of the preparation of micelle doxorubicin (DOX)-loaded chondroitin sulfate A (CSA)-disulfide bond (ss)-deoxycholic acid (DOCA) (CSA-ss-DOCA/DOX) and the mechanism of action in a tumor cell. Modified from [110].

Chondroitin sulfate is a glycosaminoglycan derived from animal cartilage. When interacting with cancer cells, chondroitin sulfate can recognize and attach to the CD44 receptor, which can significantly promote the uptake of chondroitin sulfate-based DDS by cancer cells. In addition, chondroitin sulfate exposed to degrading enzymes (such as hyaluronidase) can be rapidly degraded, resulting in the drug release of chondroitin sulfate-based DDS [107]. However, unprocessed chondroitin sulfate has excellent water solubility, and its direct use as a drug carrier will lead to low drug utilization. Studies have shown that the conjugate system formed by cross-linking with chondroitin sulfate can significantly enhance the anti-tumor activity of hydrophobic drug molecules [108]. Compared with the unprocessed chondroitin sulfate, the cross-linked chondroitin sulfate has lower hydrophilicity and better shielding effect, which can be used as a specific carrier for active drugs [21]. Combining chondroitin sulfate with CS, the amphiphilic polymer formed can self-assemble into micelles. Modified with FA and encapsulated with bortezomib, the prepared micelles have a uniform size distribution and can release drugs in response to the slightly acidic tumor microenvironment. The modification of FA gives micelles the ability to actively target tumors and also promotes the uptake of drugs by tumor cells [109]. Liu et al. designed an amphiphilic polymeric micelle on the basis of chondroitin sulfate using the self-assembly strategy (Figure 10). The reduction-sensitive chondroitin sulfate A (CSA) was combined with deoxycholic acid (DOCA) through disulfide bonds, and the therapeutic drug DOX was encapsulated into the micelle simultaneously. The micelle released DOX in response under the high concentration of endogenous GSH, realizing specific treatment of tumors [110]. In organisms, chondroitin synthase-1 (CHSY1) is an enzyme responsible for the synthesis of chondroitin sulfate, which is related to the occurrence of some tumors, especially those highly expressed in the colorectal area. The biopolymer delivery carrier combined with chondroitin sulfate has a good targeting property in the delivery of anti-tumor drugs. It could target drugs to the colon; deliver genes to the tumor tissue; and reduce the expression of CHSY1, which has a good therapeutic effect on colon cancer [111].

In recent years, various types of polysaccharides have been widely used as DDSs (Table 2). HA derived from the body itself could bind to the CD44 receptor upregulated by cancer cells, and has immunogenicity that other polysaccharides cannot match [79]. HA-mediated DDSs have the ability to actively target, which can achieve self-degradation by responding to environmental changes [80]. CS is rich in amino groups and can easily be chemically modified. CS-based DDS is prone to be endocytosed by cancer cells due to its ability to adhere to cell membranes and its low immunogenicity [87]. The molecular chain of dextran is rich in hydroxyl groups, which is convenient for chemical modification. Dextran is often used to construct drug carriers such as micelles [94] and hydrogels [93]. Alginate can chelate polyvalent metal ions to form hydrogels for drug carriers [103]. Similar to HA, chondroitin sulfate can also recognize and bind to CD44 receptor on cancer cells. Chondroitin sulfate-based DDS can be effectively taken up by cancer cells. [107]. In addition, many other polysaccharides such as pullulan [112] and heparin [113] have also been developed as DDSs, showing excellent anti-tumor properties. The development and utilization of polysaccharide-based DDSs have shown significant advantages in the field of cancer therapy.

## 5. Conclusions and Perspectives

Both toxic side effects on the human body and the difficulties in overcoming the human body’s physiological barriers of drug delivery (blood circulation, tumor accumulation, tumor tissue penetration, endocytosis, and drug release) limit the utilization of traditional chemotherapy drugs. The proposal of DDSs has opened up new prospects for the application of chemotherapy in tumor therapy. The technical support of DDSs lies in the preparation of carriers with great performance. The development of new materials or the modification of existing materials is the key to constructing efficient carriers. As abundant and renewable resources in nature, polysaccharides have been proven to be ideal materials in the construction of DDSs, which possess good biocompatibility and unique physicochemical properties. Meanwhile, the application of polysaccharides to DDSs continues to have several challenges. On the one hand, the molecular weight and structure of polysaccharides are affected by the season and environment. Some polysaccharides lack solubility in some commonly used solvents, which limits the chemical modification and drug-loading conditions. On the other hand, the form of interaction between polysaccharides and drugs is varied. The design of polysaccharide-based DDSs is still in the exploratory stage. It should be noted that many studies have simply evaluated the properties and pharmacology of DDSs through in vivo and in vitro experiments; thus, the interactions between DDSs and the human body, including absorption, distribution, metabolism, and excretion of the carrier in the human body, need to be carefully studied. The design of DDSs based on polysaccharides should be considered holistically. It is conceivable that polysaccharide-based DDSs with promising performance and therapeutic effect that are fabricated in green and effective techniques will be soon applied in clinical settings.

## Figures and Tables

**Table 1:** The commonly used functionalized types of polysaccharide molecules.

Functional Types	Modification Methods	Features	References
Modification of functional molecules	Aldehyde modification to generate Schiff base	pH-responsive capability	[21]
	Targeting ligand modification	Tumor cell targeting ability	[57,58]
Molecular chain grafting	Grafting hydrophobic segment	Amphiphilicity	[59,60,61]
Introduction of cleavable bonds	Disulfide bond	Response to the reducing microenvironment	[62,63]
	pH-sensitive groups	Respond to the weak acid microenvironment	[64]

**Table 2:** Research progress of common polysaccharide-based DDSs.

Name	Design Strategy	Functionalization Method	Characteristics of DDS	In Vivo Model	Reference
HA	Preparation of drug-loaded polysaccharide-based hydrogels (HA-based hydrogel encapsulates DNase1)	Modification of functional molecules (cholesteryl moiety and methacrylate)	CD44 receptor targeting and biodegradable	A549 cells	[84]
HA	Cross-linking between polymeric electrolyte and ion (between HA and DOX)	Modification of functional molecules (HA itself as a functional molecule)	CD44 receptor targeting	B16F10, A549, H22, and HK2 cells	[85]
CS	Constructing polysaccharide–drug conjugates (NCTD)	Modification of functional molecules (CS itself as a functional molecule)	Anti-tumor effect improved and systemic toxicity reduced	BEL-7402 cells	[89]
CS	Constructing polysaccharide–drug conjugates (DOX)	Modification of functional molecules (lactobionic acid)	pH-responsive	HepG2 and SMMC-7721 cells	[90]
CS	Self-assembly of hydrophobic polysaccharides	Introduction of cleavable bonds (disulfide bond)	Controlled release	MDA-MB-231 cells	[91]
Dextran	Constructing polysaccharide–drug conjugates (ovalbumin, OVA)	Introduction of cleavable bonds (disulfide bond)	GSH-responsive	D1 cells (DCs)	[98]
Dextran	Preparation of drug-loaded polysaccharide-based hydrogels (dexmethasone or indomethacin)	Molecular chain grafting (electroactive aniline trimer hexamethylene diisocyanate)	Electro-responsive	L929 cells	[99]
Dextran	Self-assembly of hydrophobic polysaccharides	Modification of functional molecules (carboxybetaine)	Protein antifouling	HeLa cells	[100]
Dextran	Preparation of drug-loaded polysaccharide-based hydrogels (Pr)	Modification of functional molecules (phosphate)	pH-responsive and anti-tumor effect improved	HeLa and HEp-2 cells	[101]
SA	Constructing polysaccharide–drug conjugates (1-[4-(2-aminoethyl) phenoxy] zinc (II) phthalocyanine)	Modification of functional molecules (SA itself as a functional molecule)	Tumor-associated phagocyte targeting and photodynamic therapy improved	J774A.1 and HepG2 cells	[106]
Chondroitin sulfate	Self-assembly of hydrophobic polysaccharides	Modification of functional molecules (FA)	pH-responsive and tumor targeting	A549, HCT-116, and HT-29 cells	[109]
Chondroitin sulfate	Self-assembly of hydrophobic polysaccharides	Introduction of cleavable bonds (disulfide bond)	GSH-responsive	HGC-27 cells	[110]

## Abbreviations

DDS	Drug delivery system
LRP	Living radical polymerization
ROP	Ring-opening polymerization
CMC	Carboxymethyl chitosan
OSA	Octenyl succinic anhydride
DOX	Doxorubicin
GSH	Glutathione
FA	Folic acid
OCS	Oxidized chondroitin sulfate
BSA	Bovine serum albumin
CMs	Chitosan-based microspheres
CRP	Controlled living polymerization
PCL	Polycaprolactone
PGA	Polyglycolide acid
ATRP	Atom transfer radical polymerization
HA	Hyaluronic acid
PTX	Paclitaxel
CS	Chitosan
GC	Galactosylated chitosan
NCTD	Norcantharidin
GO	Graphene oxide
REC	Rectorite
DP	Dextran phosphate
Pr	Prospidine
SA	Sodium alginate
DOCA	Deoxycholic acid
CHSY1	Chondroitin synthase-1
HCLR	HA/HAase/CS/liposome/shRNA
CD44	Cluster of differentiation 44
EPR	Enhanced permeability and retention
IAP	Silencing the inhibitor of apoptosis
CSA	Chondroitin sulfate A
